# The Role of Suppression and the Maintenance of Euthymia in Clinical Settings

**DOI:** 10.3389/fpsyg.2021.677811

**Published:** 2021-05-20

**Authors:** Emanuele Maria Merlo, Anca Pantea Stoian, Ion G. Motofei, Salvatore Settineri

**Affiliations:** ^1^Department of Adult and Childhood Human Pathology “Gaetano Barresi,” University of Messina, Messina, Italy; ^2^Carol Davila University of Medicine and Pharmacy, Bucharest, Romania; ^3^National Institute of Diabetes, Nutrition and Metabolic Diseases “N. C. Paulescu,” Bucharest, Romania; ^4^Department of Biomedical and Dental Sciences and Morphofunctional Imaging, University of Messina, Messina, Italy

**Keywords:** clinical psychology, defense mechanisms, emotional distress, euthymia, suppression

## Abstract

**Background:** Defense mechanisms serve as mediators referred to the subjects’ attempt to manage stressors capable of threatening their integrity. Mature defense mechanisms represent the high adaptive group, including suppression, which allows the subject to distance disturbing contents from consciousness. In line with general defensive intents, suppression would preserve stable mood states, as in the case of euthymia. Clinical issues usually disturb *homeorhesis*, so that the study of subjects’ suppressive tendencies would suggest possible existing relations among defense mechanisms, mood states, and clinical issues. The study highlighted the significant existing relations among factors such as suppression, euthymia, mood states, and clinical psychological phenomena.

**Methods:** The observation group was composed of 150 participants, 51 males (34%) and 99 females (66%), aged from 25 to 30 years old, with a mean age of 26.63 years old (SD = 1.51). The study was conducted through the use of measures related to subjects’ characteristics, euthymia, psychological flexibility and psychological well-being (Euthymia Scale), suppression (Suppression Mental Questionnaire), well-being (Who-5), and compassion (ProQol-5).

**Results:** The performed analyses consisted of descriptive statistics, correlations, differences, and regressions among the considered variables. Starting from the first hypothesis, SMQ factors appeared to be significantly and positively correlated with Euthymia factors, rather than Regression in the Ego service (-). In line with the previous result, significant and positive correlations emerged among SMQ and Well-being (WHO-5) variables, maintaining an inverse relation with Regression in the Ego service. Significant differences emerged between male and female groups concerning SMQ total score and rationalization, with higher male group scores. Finally, significant dependencies emerged among the selected predictors (SMQ variables) and Compassion satisfaction.

**Conclusion:** The emerged results highlighted significant relations among the considered variables so that it was possible to highlight the common directions assumed by suppression variables, well-being, and euthymia. Moreover, suppression appeared as a significant predictor with a causal role in clinical satisfaction. The results that have emerged allow us to consider defenses through an empirical perspective, useful to suggest an extension to other groups, phenomena, and conditions.

## Introduction

The maintenance of a stable mood can be influenced by different factors, including defense mechanisms whose role can be understood starting from classical contributions due to Sigmund Freud’s efforts. In these terms, the concept of a defense mechanism has appeared since 1894, regarding the origin of hysteric symptomatology and manifestations. Some relevant contributions treated the defense mechanisms referring to their origins, impact on clinical practice, and future directions. In line with Vaillant’s (1992) perspective, the theme of defense mechanisms can be considered as historically directed to modern methods, directed to the fields of clinical practice. Defense mechanisms can be considered as predominantly unconscious. Their use can refer to specific psychopathological domains, although their appearance is strongly linked to the subject’s developmental stages. According to Anna Freud (1936), their use can be considered as reversible, strictly linked to subject’s structure and environmental conditions.

Current research considers the possibility of involving defense mechanisms in empirical terms. It is always more fruitful to deal with high validity instruments adherent to classical contributions and new perspectives. In 1994, APA defined the defense mechanisms as unconscious operations, whose role is linked to protecting the individual from thoughts, feelings, internal, or external conflicts. This definition respects the theme of defense, since its use is strongly related to the use of defenses in avoiding conflicts properly referred to internal/external stressful issues. The anguish derived from these types of disputes puts the subject in a condition useful to react, depending on structure.

The research on defense mechanisms allowed Vaillant (1977) to propose a hierarchical model considering defenses organized at different levels so that archaic defenses were distinguished from neurotic and high adaptive/mature defenses. Different levels were provided and involved in developing empirical instruments useful to compare theoretical issues to research, clinical practice, and future possibilities (Perry and Henry, 2004).

As suggested by recent research (Di Giuseppe et al., 2020a), the effort to develop valid instruments was referred both to clinical and healthy populations, so that it would be possible to keep the transversal relevance of defenses (Merlo et al., 2021). In literature, many studies expressed the need to extend the study of defensive patterns to different clinical conditions and developmental stages (Di Riso et al., 2015; Di Giuseppe et al., 2019; Gugliandolo et al., 2020). The stressors’ incidence on the onset of lesions and functional maladjustment, can derive from specific psychological factors (Lingiardi et al., 2010; Perry et al., 2015; Porcerelli et al., 2017; Gangemi et al., 2018; Catalano et al., 2019; Conversano, 2019; Fiegl et al., 2019; Kelly et al., 2019; Martino et al., 2019, 2020a,2020b,2020c; Merlo, 2019; Vita et al., 2020). Clearly, the possibility to obtain data from research studies dedicated to the mentioned themes took place through the efforts to operationalize the theoretical basis, constructing empirical instruments, as in the case of Perry’s DMRS ([Bibr B57][Bibr B57]; Di Giuseppe et al., 2020b), of Bond’s DSQ-40 (Bond et al., 1983; Farma and Cortinovis, 2000; Perry and Bond, 2012) and other valuable instruments.

In these terms, according to Berney et al. (2014), clinical and empirical aspects of defense mechanisms provide knowledge about affective dynamics and tendencies occurring in all individuals, both clinical and non-clinical populations. In their hierarchical organization, defense mechanisms present different features, so that archaic defenses differ from neurotic and adaptive ones. Vaillant (1994) therefore introduced humor, sublimation, suppression, altruism, and anticipation in the IV category, respectively, named mature defenses. In terms of adaptive tendencies and dynamics, repression has been considered as a high adaptive defense (Metzger, 2014), included among mature defenses and considered by Vaillant as follows: “*When used effectively* [.] *suppression is analogous to a well-trimmed sail*” (2000, p.94). Suppression can be considered as a predominantly conscious defense mechanism, whose use is close to the subject’s conscious need to avoid disturbing contents. Although the role of this defense can be considered as similar to repression, this last represents a predominantly unconscious dynamic. The main difference between these two defense mechanisms is based on the level of subject’s consciousness during the use of the defenses. Through suppression, the individual overcomes internal/external issues, replacing the disturbing contents with more adaptive themes and tasks.

When considering the role of defense mechanisms in avoiding anguish and maladjustment, it is fundamental to mention what can be regarded as directly deriving from their adaptive or excessive use. Affective dynamics result as indirectly involved but still present. In these terms, although defense mechanisms act in the regard to affectivity and representations, their role does not disappear. Most of the psychosomatic issues in fact represent the final stage of excessive defensive tendencies. Beside displacing disturbing contents from consciousness, regressions to fantasy are often included (Kris and Kaplan, 1952; Kris, 1952a,b,c; Knafo, 2002) defined this tendency as “Regression in the Service of the Ego,” in order to describe short regressions useful to avoid anguish and to manage representations without reality limits. Thus, empirical research demonstrated how alexithymia does not correspond to the absence of emotions, rather than in a lack of mentalization. Some concepts tend to attract more attention depending on literature trends, but understanding basic phenomena producing comprehensive definitions is fundamental (as in the case of resilience and well-being). Over the years, the concept of well-being has undergone numerous changes and extensions that have led to a broader and more complete vision of the term, no longer focused on the absence of pathologies, suffering, or discomfort, but on a condition of balance between the person and the environment. Connected to this construct is the concept of *euthymia*.

In the psychiatric literature, the term euthymia essentially indicates the lack of significant distress. In psychology, “euthymia” shows the typical mood of the non-depressed individual, who experiences a serene or neutral mood. In clinical settings, euthymia is often defined only in negative terms as the absence of symptoms related to mood, neglecting the positive aspects of curing. In 1958, psychologist Marie Jahoda created a model of psychological well-being made up of various dimensions, such as regulating behavior (internal), environmental control, satisfying and positive relationships with others, and the degree of personal growth, self-realization, and self-acceptance.

Jahoda also underlines a characteristic linked to the concept of euthymia: “integration,” a balance of psychic forces that corresponds to the concept of “psychological flexibility,” an ability useful to maintain individual balance and resist stress (greater resilience and tolerance to frustration). Based on these terms, some research contributions offered the possibility to operationalize the concepts in order to reach empirical validity and reliable instruments. Starting from these concepts, (Guidi and Fava, 2020; Fava and Bech, 2016) pointed out that the purpose of clinical evaluation is to explore the presence of positive affectivity, psychological well-being, and their interactions with the course of symptoms. To analyze these features in an integrative way, a clinimetric perspective is necessary.

The term clinimetric indicates an area interested in measuring clinical problems with no place in the standard clinical taxonomy. These problems include types, severity and sequence of symptoms, disease progression rate (staging), severity of the comorbidity, functional capacity problems, reasons for medical decisions (e.g., therapeutic choices), and many other aspects of daily life, such as well-being and distress. Directly related to this path, several research contributions arose from the commitment to measure empirically clinical issues through a cinimetric perspective (Carrozzino et al., 2019; Guidi and Fava, 2021), with a look toward well-being, psychopathology and psychotherapy (Fava et al., 2017).

Considering the figures mentioned above, we stated four hypotheses to highlight common directions, differences, and dependencies among the included phenomena (alexithymia, well-being, suppression, and compassion). In detail, the following paragraph describes the relationships and implications of potential variables.

### The Current Study

Four hypotheses were stated in order to allow the relations to emerge according to the methodology.

Hypothesis 1: We hypothesize that suppression assumes similar directions with euthymia. In particular, we hypothesize positive correlations with euthymia, rather than Regression in the Ego service.
Hypothesis 2: We hypothesize coherent directions assumed by suppression and well-being, except for Regression in the Ego service.
Hypothesis 3: We hypothesize significant differences between male and female groups concerning Euthymia, Suppression, and Well-being.
Hypothesis 4: We hypothesize the existence of significant dependencies among suppression variables and clinical commitment variables, highlighting the causal role of suppression linked to adaptation.

## Materials and Methods

### Procedure and Participants

The observation group consisted of 150 healthy participants, 51 males (34%) and 99 females (66%). The age of the participants included in the study was between 25 and 30 years old, with a mean age of 26.63 years old (SD = 1.51). The research was carried out at the University of Messina, Italy with the aim of exploring clinical psychological issues strictly related to clinical practice. All participants were involved in clinical assistance.

Every participant fully completed the questionnaires, including information regarding their activities, studies, gender, and age. Each participant fully completed a checklist referred to health status, in order to be considered admissible for the final group. Health subjects were selected in order to complete the questionnaire. The checklist was both referred to psychological and physical domains. The compilation of the questionnaires was an online form due to the current COVID-19 pandemic.

Before adhering to informed consent, each participant was informed about the anonymous nature of the methods of data processing, as required by the procedures of the ethical committee evidenced by the approval (University of Messina COSPECS Ethical Committee, Ethical committee number: COSPECS_14_2020).

### Statistical Analysis

The data were expressed as a mean and a standard deviation, and the categorical variables as number and percentage.

According to the study hypotheses, the “Spearman test” was applied to evaluate the correlations among variables of the following instruments.

The Student’s *t* test compared gender groups, referring to euthymia, well-being, and suppression.

Multivariate linear regression was used to assess each of the ProQol clinical outcomes’ dependence on a set of independent predictors (SMQ Total Score and related factors).

Statistical analyses were performed using SPSS 26.0 for the Window package.

A *P* value smaller than 0.050 was considered to be statistically significant.

### Instruments

#### Suppression Mental Questionnaire

The Suppression Mental Questionnaire (Settineri et al., 2019a, b) consists of 18 items assessing the use of the mature defense mechanism named suppression, based on a five-point Likert scale. SMQ is a self-report instrument validated in both paper-and-pencil and app version, whose validation highlighted the existence of three main factors, namely, Repressive function, Regression in the service of the Ego, and Rationalization.

According to classical literature, suppression is meant as the capacity to banish disturbing contents from consciousness. This capacity is related to a consistent, conscious effort, useful to let the subject direct his/her resources toward adaptive activities avoiding anguish deriving from disturbing contents.

The validation of the instrument showed good sampling adequacy (K.M.O. = 0.648) and the following Cronbach coefficients: Repressive function = 0.742; Regression in the service of the Ego = 0.804; Rationalization = 0.698. In order to describe the inner structure of the instrument, the following items appeared as belonging, respectively, to each factor: Repressive function composed by items 3, 4, 7, 8, 10, 14, 15, 16, 17, and 18; Regression in the service of the Ego, items 5, 6, 9, 11, and 12; Rationalization, items 1, 2, 7, and 13. In line with the paper-and-pencil version, the instrument’s app adaptation provided for the following alpha coefficients: 0.74–0.73 for the first factor, 0.80–0.77 for the second, and 0.70–0.76 for the third one.

### WHO-5

World Health Organization (Five) Well-being Index [World Health Organization (WHO), 1998], composed of five items assessing well-being, evaluated using a six-point Likert scale from 5 (always) to 0 (never). According to the validation study [World Health Organization (WHO), 1998] and with several other research items (Topp et al., 2015), the scale appeared as a valid instrument useful for assessing subjects’ well-being. As a generic scale, its properties are related to the possibility to evaluate mental well-being through a limited number of items (Hall et al., 2011; Bech, 2012).

### Euthymia Scale

Through their clinimetric analysis, Carrozzino et al. (2019) showed that a good definition for Euthymia (provided by Fava and Bech, 2016) has been considered as the preliminary step to build up a scale useful for the evaluation of the selected phenomenon. Fava and Bech (2016) incorporated Jahoda’s definition of euthymia Jahoda’s (1958) to develop a self-report rating scale named Euthymia Scale. In these terms, the purpose regarded the absence of affective disorders and the presence of psychological flexibility and resistance to stressors. Their clinimetric study, directly linked to this definition, provided two dimensions: Psychological flexibility and Psychological well-being. The analysis showed the scale’s validity and its two dimensions through two main clinimetric parameters, known as scalability and incremental validity (respectively, assessed through Mokken and hierarchical linear regression analyses). The study on healthy subjects produced the following indexes, referred to Mokken analysis: 0.25 for the total score of the 10-item Euthymia Scale, 0.28 for Psychological flexibility, and 0.30 for Psychological well-being.

### ProQol-5

The Professional Quality of Life Scale (ProQol-5) is a self-report instrument dedicated to studying adaption and maladjustment deriving from clinical practice. The scale was validated by Stamm (2005, 2009) and adapted in Italian by Palestrini et al. in Palestrini et al., 2009. The scale consists of 30 items supported by a five-point Likert scale.

The factorial analysis showed three different factors, respectively, Compassion satisfaction, Burnout, and Secondary traumatic stress. Each factor consists, respectively of the following items: Factor 1, items 3, 6, 12, 16, 18, 20, 22, 24, 27, and 30; Factor 2, items 1^∗^, 4^∗^, 8, 10, 15^∗^, 17^∗^, 19, 21, 26, and 29 (^∗^ for inverted scores); Factor 3, items 2, 5, 7, 9, 11, 13, 14, 23, 25, and 28. Reliability indexes provided by the authors were as follows: Compassion Satisfaction, alpha scale reliability = 0.88; Burnout, alpha scale reliability = 0.75; Secondary traumatic stress, alpha scale reliability = 0.81.

## Results

Descriptive statistics (mean and the standard deviation) are reported in Table 1 in order to highlight the presence of considered phenomena.

**TABLE 1:** Descriptive statistics for study variables.

	**Mean**	**Standard deviation**
Years of Study	14.41	2.30
Euthymia Scale	6.70	2.19
Euthymia Scale Psychological Flexibility	3.58	1.22
Euthymia Scale Psychological Well-Being	3.12	1.43
SMQ Total Score	52.52	8.42
SMQ Repressive function	24.56	6.58
SMQ Regression in the service of the Ego	18.21	4.07
SMQ Rationalization	13.71	2.57
WHO-5	14.34	4.02
Compassion satisfaction	39.84	5.41
Burnout	23.26	4.98
Secondary traumatic scale	23.90	6.05

Hypothesis 1:

Hypothesis 1 (Table 2) concerned the directions assumed by the considered phenomena in correlational terms so that the factors of Euthymia and Suppression Questionnaire were involved. As it is possible to assume, according to the values reported in Table 2, all correlational relationships emerged among SMQ factors, Euthymia Scale Total Score, and Euthymia Scale Psychological Flexibility were significant. Six of the relationships, as mentioned earlier, emerged as significant and positive, two as significant and inverse. In detail, the positive relations were referred to Euthymia Total Score and Psychological Flexibility with SMQ Total Score, SMQ Repressive function, and SMQ Rationalization. In our perspective and line with the considered literature, Suppression appeared to assume the same direction of Euthymia, highlighting a positive relation. It is meant as a positive role deriving from mature defense and adaptation mechanisms, in line with classical studies and recent empirical research contributions. Suppression appeared to be correlated significantly and positively, confirming the role of high mature defenses on mood states.

**TABLE 2:** Correlation coefficients among SMQ and Euthymia Scale variables.

	**Euthymia Scale Total Score**	**Euthymia Scale Psychological Flexibility**	**Euthymia Scale Psychological Well-Being**
SMQ Total Score	0.321**	0.378**	0.187**
SMQ Repressive function	0.468**	0.474**	0.315**
SMQ Regression in the service of the Ego	−0.238**	−226**	–0.159
SMQ Rationalization	0.297**	0.432**	0.110

*p < 0.05 (two-tailed); **p < 0.01 (two-tailed). Bold values were the significant values.

Concerning Regression in the Ego service, it appeared to be significantly and inversely correlated to Euthymia Total Score and Psychological Flexibility showing as the regression to phantasies and phantasmatic atmospheres is closely related to archaic-type functioning built on the necessity to avoid reality limitations and lows. In compliance with the performed analyses, suppressive tendencies appeared as mainly directed to maintaining a stable mood. Concerning Psychological Well-Being (Euthymia Scale’s second dimension), the two significant and positive relations that emerged were referred to SMQ Total Score and Repressive functions. In these terms, the significant correlations that emerged highlighted the directions assumed by the variables so that suppressive necessities showed a higher propensity to take the same direction of stable mood maintenance. In clinical terms, suppression had a crucial role in favoring the adaptation of the subjects. The correlational analysis provided for relevant significant relations, so that increasing suppressive tendencies corresponded to higher levels of euthymia. In terms of mood balance and adhering to classical and empirical literature, suppression demonstrated his adaptive direction.

Hypothesis 2:

Hypothesis 2 (Table 3) referred to the occurring relationships among suppressive tendencies and subjects’ well-being. The used instruments provided for different factors involved. Correlational analyses were performed in order to highlight common directions among the considered phenomena, so that significant relations emerged among SMQ Total Score, SMQ Repressive function, SMQ Regression in the service of Ego, and WHO-5 Well-being scale. No significant association emerged regarding SMQ Rationalization.

**TABLE 3:** Correlation coefficients among SMQ and WHO-5 factors.

	**WHO-5**
SMQ Total Score	0.260**
SMQ Repressive function	0.365**
SMQ Regression in the service of the Ego	−0.181*
SMQ Rationalization	0.149

*p < 0.05 (two-tailed); **p < 0.01 (two-tailed). Bold values were the significant values.

Two of the three significant relations were positive, rather than SMQ Regression in the service of the Ego. No significant relation emerged concerning SMQ Rationalization. The first two significant and positive relations showed the same direction of suppressive tendencies and well-being, in line with previous results referred to euthymia.

Moreover, in the previous correlational analysis, Regression in the service of the Ego emerged significantly but inversely related to well-being. Generally, as for euthymia, the increase of well-being corresponded to the rise of suppression. This fact appears as confirmatory of the theoretical aspects of mature defense mechanisms. As emerged with reference to mood balance and euthymia, the increase of the suppressive tendency corresponded to higher scores in the well-being domain. This fact highlighted how in clinical terms defenses maintain their role, in this case with direct reference to adaptation.

Hypothesis 3:

Hypothesis 3 (Table 4) refers to the emergence of possible significant differences between the two groups, respectively, male and female subjects. The analysis performed through the Student’s *t* test involved parametric variables, meant as factors related to Euthymia, SMQ Total Score, Repressive function, Regression in the Ego service, Rationalization, and WHO-5 well-being. Significant differences emerged regarding SMQ Total Score and Rationalization. Considering the highest mean scores, they were both referred to male groups, highlighting a greater score in the use of mature defense mechanisms such as Suppression, with rationalization tendencies.

**TABLE 4:** Comparison between male and female groups.

**Variables**	**Male**	**Female**	***p* value**
Euthymia Scale	6.88 ± 2.10	6.61 ± 2.24	0.475
SMQ Total score	54.43 ± 6.47	51.54 ± 9.14	0.027*
SMQ Repressive function	25.86 ± 5.46	23.88 ± 7-02	0.060
SMQ Regression in the service of the Ego	18.17 ± 3.86	18.23 ± 4.20	0.935
SMQ Rationalization	14.41 ± 2.42	13.35 ± 2.58	0.015*
WHO-5	14.82 ± 3.66	14.10 ± 4.19	0.279

*p < 0.05. Bold values were the significant values.

Hypothesis 4:

Hypothesis 4 (Table 5) was aimed at highlighting the emergence of possible dependencies among the set of predictors, namely, SMQ Total Score, SMQ Repressive Function, SMQ Regression in the service of the Ego, SMQ Rationalization, and three dependent variables contained into ProQol-5 Scale, specifically Compassion satisfaction, Burnout, and Secondary traumatic stress.

**TABLE 5:** Multivariate linear regressions analysis.

	**SMQ Total Score**		**Repressive function**		**Regression in the service of the Ego**		**Rationalization**	
	**B (CI)**	***P***	**B (CI)**	***P***	**B (CI)**	***P***	**B (CI)**	***P***
Compassion satisfaction	−1.90 (−3.36; −0.438)	0.011*	2.07 (0.661; 3.49)	0.004*	2.13 (0.680; 3.59)	0.004*	1.36 (0.109; 2.63)	0.033*
Burnout	1.11 (−0.260; 2.48)	0.112	−1.22 (−2.55;0.105)	0.071	−0.857 (−2.22;0.511)	0.218	−0.857 (−2.01;0.511)	0.165
Secondary traumatic stress	−0.902 (−2.51;0.710)	0.271	0.720 (−0.843; −2.28)	0.364	1.36 (−0.244; 2.97)	0.096	0.691 (−696; 2.07)	0.326

B = Beta coefficient; CI = confidence Interval; *p < 0.05 was considered as significant for the multivariate linear regression analyses.

The significant dependencies were referred to Compassion satisfaction, with reference to the whole set of independent variables.

The emerged dependence relationships highlighted the role of defensive paths on clinical commitment in terms of the usefulness of high adaptive defenses. In particular, repression appeared as directly responsible for SMQ factors’ increase.

These data have placed the present findings in line with previously performed analyses and research, referring to the employment of defense mechanisms to reach an adaptive tendency and stable object relations in clinical terms. Finally, in clinical terms, the high adaptive role of suppression took place with direct reference to causal relations. In the current study, suppression assumed coherent directions with mood balance and well-being. In causal terms, suppression demonstrated to directly influence clinical satisfaction.

## Discussion

The present study highlighted the existing relations among relevant phenomena such as defense mechanisms, mood states, and clinical issues. In particular, the study hypotheses included the abovementioned phenomena in order to study their directions, differences, and dependence relations.

Starting from the considered defense mechanism, suppression emerged as a consistent way to manage internal/external stressors, as in the case of adaptation, pathological psychosomatic issues, and medical conditions due to psychological phenomena (Warnes, 1982; Cramer, 2000; De Burge, 2001; Szwec, 2018; Settineri et al., 2019a; Conversano et al., 2020b; Conversano and Di Giuseppe, 2021).

In particular, the first statistical analysis was performed regarding the existing relations among suppression and euthymia, explaining significant associations. The protective role of suppression appeared as linked to euthymia, so that higher levels of suppression corresponded to higher scores related to euthymia. Therefore, suppression represents a conscious dynamic steaming from of the need to remove disturbing contents from cognition. We are aware of this fact in the subject’s attempts to reach adaptation, where personal facts, representations, and adverse affectivity are spaced out in order to pursue tasks. Distress arising from inappropriate emotions, feelings, and images is thus avoided by the mechanism of suppression.

The significant and positive relations were referred to general terms (meant as suppressive functioning) and strictly related factors. In opposite terms, regression to fantasy emerged aversive instead of the maintenance of positive mood and affective stability.

The link between defensive structure, coping, and mood regulation appears more and more precise, directly supported by recent literature (Brockman et al., 2017; Compas et al., 2017; Schäfer et al., 2017; Weissman et al., 2019). The need to avoid unsatisfying and adverse consequences due to representations and emotions accounts for one of the main themes in defenses. In these terms, our study described a continuity with previously emerged studies.

As a consequence of this emerged result, well-being appeared as significantly associated with suppression. Even in this case, Regression in the service of the Ego occurred as significantly and inversely directed. In most of the classical works, it appeared as mainly related to creativity and action, close to fantasies and regressive moves (Wild, 1965; Fitzgerald, 1966; Bush, 1969; Knafo, 2002), in our case far from rational and adaptive behaviors tending to consider needs strictly related to reality.

In our experience, the suppressive tendency was shown as the adaptation process stems as strong as directed to adjustment. Higher levels were detected in male subjects, referring to the emerged significant differences, statistically performed in our analysis.

Recent literature highlighted how subject structures influence the course of cure (Eglinton and Chung, 2011; Lomas et al., 2019; Conversano et al., 2020a; Kinsella et al., 2020), with particular reference to compassion (McNally et al., 2019; Merlo et al., 2020a, b).

In our results, dependence relations emerged concerning compassion satisfaction and all suppression factors. Even in this case, the significant dependences highlighted the role of suppression with reference to the opportunity to experience compassionate clinical circumstances (Zeidner et al., 2013; Ivicic and Motta, 2017; Singh et al., 2020). The role of compassion is currently emerging as a high influencing factor, both related to clinicians and patients and extended to several conditions such as psychopathology, therapy, mental health, image concerns, neurovegetative phenomena, and general health outcomes (Mincă et al., 2013; Rosa et al., 2019; Carter et al., 2021; Kim et al., 2021; McKay and Walker, 2021; Turk et al., 2021).

From our results, it was possible to show how phenomena treated through different models can be analyzed in terms of assumed directions and dependencies. Both in terms of defense mechanism and mood states, it was possible to highlight how mature defenses intervene in mood states to manage internal/external stressors deriving from clinical settings and practice. With particular reference to mood states, euthymia appeared significantly directed in the same direction of mature defensive processes.

This fact highlights a strong continuity with classical models and current empirical research on defense mechanisms, extended to the other main direction related to consciousness and coping. Our results suggested being compliant with current research, involving instruments of recent development, adherent to confirming previous speculative research through objective methods. Finally, the possibility offered by empirical research in our perspective represents the link between previous research and current needs to import, discuss, confirm, and possibly disconfirm data steamed from historical periods in which, from the beginning, the aim of reaching intersubjective validity was not neglected.

## Implications of the Study

Current research in the clinical field presents the need to improve empirical studies, involving and exporting classical clinical themes into valid tools. This need can be extended to the role of defense mechanisms and the maintenance of stable mood so that the current study provided empirical research based on suppression and euthymia.

Through the use of recently validated instruments, high adaptive defense and euthymia have been examined in terms of correlations, differences, and dependencies. Through the performed analyses, several significant relations emerged, highlighting congruent directions, dependencies, and differences useful to understand the role of suppression regarding mood.

According to the used instruments, the emerged results would serve as a previous basis useful to extend the results on other samples to confirm and discuss further developments. The aim to detect existent relations must be extended to further samples and clinical conditions, close to the aim of deepening defenses’ impact on mood stability. The emerged results were strictly related to clinical psychological dynamics, highlighting how the use of mature defense assumed coherent directions with mood balance and well-being, beside specific gender differences. In clinical psychological terms, these data represent relevant information, useful to understand how conscious phenomena bring the subjects closer to adaptive dynamics. In particular, a clear reference to satisfaction emerged, demonstrating the adaptive role of certain defenses in increasing assistance quality and efficacy.

## Limitations and Conclusion

The present study has several limitations highlighting the need to support the emerged results through further research. The considered subjects were involved in clinical settings, so it would be necessary to extend the number of participants to better reflect the population. Despite the emergence of significant relations, the data should be extended and compared with more extended samples.

Moreover, the number of female subjects was higher than males. This fact recalls the need to reach a gender sample balance useful to better compare groups. The participants’ age ranged from 25 and 30 years old, suggesting the purpose to include other age groups.

Although these references constitute limitations, the study was aimed at improving knowledge about the considered phenomena. This cross-sectional study can be regarded as an example of a knowledge extension on the themes mentioned earlier and clinical issues.

## Data Availability Statement

The raw data supporting the conclusions of this article will be made available by the authors, without undue reservation.

## Ethics Statement

The studies involving human participants were reviewed and approved by Ethical Committee, Department of Cognitive Sciences, Psychology, Educational and Cultural Studies (COSPECS), University of Messina, Italy. The patients/participants provided their written informed consent to participate in this study.

## Author Contributions

EM made a significant contribution to design the research study, administer the protocol, draft the manuscript, revise it critically, perform the statistical analysis, and provide data interpretation. IM and AS made a significant contribution to design and revise the research study, and SS gave the final approval. All authors listed have made a substantial, direct and intellectual contribution to the work, and approved it for publication.

## Conflict of Interest

The authors declare that the research was conducted in the absence of any commercial or financial relationships that could be construed as a potential conflict of interest.
